# Estimating the efficacy of symptom-based screening for COVID-19

**DOI:** 10.1038/s41746-020-0300-0

**Published:** 2020-07-13

**Authors:** Alison Callahan, Ethan Steinberg, Jason A. Fries, Saurabh Gombar, Birju Patel, Conor K. Corbin, Nigam H. Shah

**Affiliations:** 1 Center for Biomedical Informatics Research, School of Medicine, Stanford University, Stanford, CA USA; 2 Department of Pathology, School of Medicine, Stanford University, Stanford, CA USA

**Keywords:** Viral infection, Epidemiology

## Abstract

There is substantial interest in using presenting symptoms to prioritize testing for COVID-19 and establish symptom-based surveillance. However, little is currently known about the specificity of COVID-19 symptoms. To assess the feasibility of symptom-based screening for COVID-19, we used data from tests for common respiratory viruses and SARS-CoV-2 in our health system to measure the ability to correctly classify virus test results based on presenting symptoms. Based on these results, symptom-based screening may not be an effective strategy to identify individuals who should be tested for SARS-CoV-2 infection or to obtain a leading indicator of new COVID-19 cases.

## Introduction

There is substantial interest in developing symptom-based screening to prioritize who should be tested for SARS-CoV-2 infection and to establish symptom-based surveillance to provide an early indicator of new COVID-19 cases^1–3^. However, the degree to which presenting symptoms are reliable indicators of SARS-CoV-2 infection is unknown^4^. Therefore, it is crucial to determine whether symptom-based screening to prioritize testing is feasible. To assess the feasibility of using symptom-based screening to assign a probability of SARS-CoV-2 infection, we first quantified the ability to correctly predict results of tests for common respiratory viruses observed to frequently co-infect patients positive for SARS-CoV-2 at Stanford Health Care^5^, using symptoms mentioned in clinical notes at the time of the test order. After establishing a baseline for the performance of machine learning models to correctly classify common respiratory virus infections^6^, we then trained a similar model for SARS-CoV-2 test results^7^.

## Results and discussion

### Performance of models to predict respiratory virus test results

For the respiratory viruses examined, area under the receiver operator curve (AUROC) on the test set ranged from 0.60 to 0.77 (Table 1). Two non-SARS-CoV-2 viruses (influenza type A, and RSV) were moderately predictable given presenting symptoms. For example, mentions of *coughing*, *wheezing* and *rhinorrhea* were features with high importance for the RSV model. However, SARS-CoV-2 and the remaining common respiratory viruses (adenovirus, rhinovirus, metapneumovirus and parainfluenza) were not highly predictable, with average AUROCs below 0.70.

**Table 1:** AUROCs of logistic regression models for each respiratory virus with 95% confidence intervals .

Virus	AUROC
Adenovirus	0.68 (0.60–0.76)
Influenza virus A	0.73 (0.68–0.77)
Metapneumovirus	0.64 (0.57–0.71)
Parainfluenza virus	0.60 (0.53–0.68)
RSV	0.77 (0.73–0.80)
Rhinovirus	0.62 (0.58–0.66)
SARS-CoV-2	0.64 (0.49–0.79)

These results suggest that, for both SARS-CoV-2 and other commonly diagnosed respiratory viral infections, the presenting symptoms at the time of the test order may not provide sufficient information to correctly classify whether a given patient will test positive for that virus. Prior studies of presenting symptoms and case definitions for influenza found that information on presenting symptoms alone is not sufficient to accurately diagnose influenza, or distinguish it from other influenza-like illnesses^8–12^, and our results support this finding. Though our Influenza Virus A model had one of the higher AUROCs we observed, it is not sufficient for use in a clinical setting.

### Limitations

There are several limitations to this work. Firstly, we did not include information on the duration of reported symptoms as features in our models, because duration is often not reliably described in clinical notes and therefore difficult to ascertain^13^. The sensitivity and specificity of SARS-CoV-2 PCR tests is highest in the first few days that symptoms present^14^. It is possible that the data used in our analysis included patients tested later in the course of SARS-CoV-2 infection, which would diminish classifier performance. Secondly, the number of SARS-CoV-2 positive cases in our data was small, which is fortunate for our population’s health, but creates a sample size limitation which can adversely impact machine learning model performance. Lastly, the prevalence of positive cases in those tested for SARS-CoV-2 infection is dependent on the health system’s protocols used to decide whether to test a patient; this may impact the applicability of our findings as testing protocols evolve.

We note that clinicians’ knowledge of the signs and symptoms of COVID-19 are rapidly evolving, such as recent findings that a substantial fraction of patients experience gastrointestinal symptoms^15^ and dermatologic symptoms^16^, and the prevalence of these presentations are still being characterized. The CDC has recently updated their list of COVID-19 related symptoms to include loss of taste or smell, headache, and chills with fever^17^. Documentation of such emerging symptoms will increase in the clinical notes of tested patients. Therefore, as part of Stanford Health Care’s response to COVID-19, we continue to collect data on patients tested for SARS-CoV-2 and to profile their presenting symptoms^7,18^. Doing so will allow us to assess the effect of improved symptom characterization and additional data on the performance of models to identify SARS-CoV-2 infections in an ongoing manner.

### Summary

In summary, our current findings indicate that symptom-based screening may not be an effective strategy to quantify an individual’s likelihood of having COVID-19. The non-specific nature of the symptoms, and the fact that co-infections with other respiratory viruses are common^5^, might limit the utility of symptom-based screening strategies to prioritize testing and the use of symptom surveys as a leading indicator of new COVID-19 cases in a region^1,19^.

## Methods

### Patient cohort selection

Patients included in our study were those tested for adenovirus, influenza virus A, metapneumovirus, parainfluenza virus, respiratory syncytial virus (RSV), rhinovirus, and SARS-CoV-2 (Table 2). Historical data on patients tested for other respiratory viruses were collected between September 2010 and October 2019, and included virus tests ordered as part of respiratory virus panels as well as tests ordered on their own. Data on patients tested for SARS-CoV-2 were collected up to March 30, 2020, and also included results of tests ordered as part of respiratory pathogen panels^5^. Only the contents of the emergency department or urgent care note associated with the order of a patient’s virus test was used as input for training the models, in order to emulate the information that would be available in a real-life usage setting.

**Table 2:** Total number of patients and number of positive and negative test results used to develop models for each virus test.

Virus	Patients	Positive tests	Negative tests
Adenovirus	10,911	218	10,693
Influenza virus A	8642	980	7662
Metapneumovirus	9504	360	9144
Parainfluenza virus	9507	351	9156
RSV	13,525	1059	12,466
Rhinovirus	8305	1387	6918
SARS-CoV-2	895	64	831

### Feature engineering

Each patient’s test-associated note was processed using a rule-based NLP pipeline^20^ to extract mentions of medical concepts. Extracted concepts were classified to filter out negated terms, to identify relative timing of the concept (e.g. a current condition or past condition), and to identify note sections, in order to filter out concepts that were not noted at initial observation but added later in the course of documentation. All concepts were derived from the 2018AA SNOMED CT US vocabulary with the semantic group DISORDER^21^, maintained as part of the National Library of Medicine Unified Medical Language System.

Extracted concepts were encoded as binary variables to indicate their presence or absence in a given patient’s clinical note, after filtering to keep only the non-historical, positive mentions about the patient and restricting to signs and symptoms based on SNOMED CT US semantic types. The outcome was whether the virus test returned positive or negative for infection with the tested pathogen.

### Model training and evaluation

For respiratory viruses other than SARS-CoV-2, we trained logistic regression models on a randomly selected 80% sample of patients’ note-derived data and tested their performance on the remaining 20%. We calculated 95% confidence intervals using bootstrapping on the testing set. We evaluated performance using the AUROC, which is a measurement of a model’s ability to distinguish positive and negative test results. For SARS-CoV-2, because we were not able to use a fixed test set due to the limited sample size of tested patients, we performed tenfold cross validation and estimated uncertainty with a t-distribution confidence interval. Hyperparameters were tuned using cross validation on the training set. This study was approved by the Stanford University Institutional Review Board, and this approval included a waiver of informed consent due to the retrospective nature of the study.

### Reporting summary

Further information on research design is available in the Nature Research Reporting Summary linked to this article.

## Supplementary information

Reporting Summary

## Data Availability

www.tinyurl.com/symptom-profile contains a summary of the top 50 most frequent clinical observations extracted from the clinical notes of patients tested for SARS-CoV-2 at Stanford Health Care and will be updated monthly through to September 2020.

## Code availability

Feature engineering, model training, and evaluation were conducted in Python using scikit-learn version 0.22.1. Code is available upon request from the corresponding author.
